# A rare simultaneous occurrence of appendiceal diverticulitis and non‐perforated peptic ulcer mimicking abdominal peritonitis symptoms

**DOI:** 10.1002/ccr3.5780

**Published:** 2022-04-27

**Authors:** Maziar Moayerifar, Hossein Torabi, Kasra Shirini, Yalda Ashoorian

**Affiliations:** ^1^ 37554 Department of General Surgery, Poursina Medical and Educational Center Guilan University of Medical Sciences Rasht Iran; ^2^ Department of General Surgery Iran University of Medical Science Tehran Iran; ^3^ Department of Pathology and Laboratory Medicine Guilan University of Medical Science Rasht Iran

**Keywords:** appendiceal diverticula, appendiceal diverticulitis, laparotomy, peptic ulcer, peritonitis

## Abstract

Appendiceal diverticulitis is an infrequent disease that can mimic other diseases’ symptoms or cause different symptoms because of its various complications. However, one of the most frequent complications of this disease is a perforation that can lead to other serious problems such as peritonitis. This complication can threaten a patient's health condition. In this article, a male patient presented with abdominal pain and was admitted to the surgical ward with suspicion of a perforated peptic ulcer. However, more investigation showed a simultaneous occurrence of peptic ulcer and perforated appendiceal diverticulitis that cause peritonitis symptoms.

## Graphical abstract

Appendiceal diverticulitis is a rare condition that can mimic other diseases’ symptoms or cause different symptoms because of its various complications and it should be considered as a serious and important differential diagnosis.

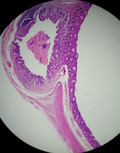

## 1 INTRODUCTION

The first person who described Appendiceal diverticulitis was Kelynack, a pathologist in the 19th century. He described it as "a greatly distended appendix, totally shut off from the cecum, having two distinct diverticular processes directed between the folds of the mesentery" and classified it into congenital and acquired types.^1^, ^2^ It is a rare condition with a prevalence incidence of 0.014%–1.9%.^2^, ^3^, ^4^ Studies showed that despite the similarities between appendiceal diverticulitis and acute appendicitis, the prevalence of perforation in appendiceal diverticulitis is three to four times higher which increases the risk of abdominal peritonitis. Also, it can be synchronous neoplasms such as carcinoids, mucinous adenomas, and adenocarcinomas, or it can mimic other diseases’ symptoms. So, the preoperative diagnosis of this disease is challenging and essential.^2^, ^5^ As regards this issue, a patient with generalized abdominal peritonitis symptoms, without any specific appendicitis symptoms, presented in this article and finally discovered appendiceal diverticulitis as the main reason but mimicking abdominal peritonitis due to perforated peptic ulcer.

## 2 CASE PRESENTATION

**FIGURE 1:**
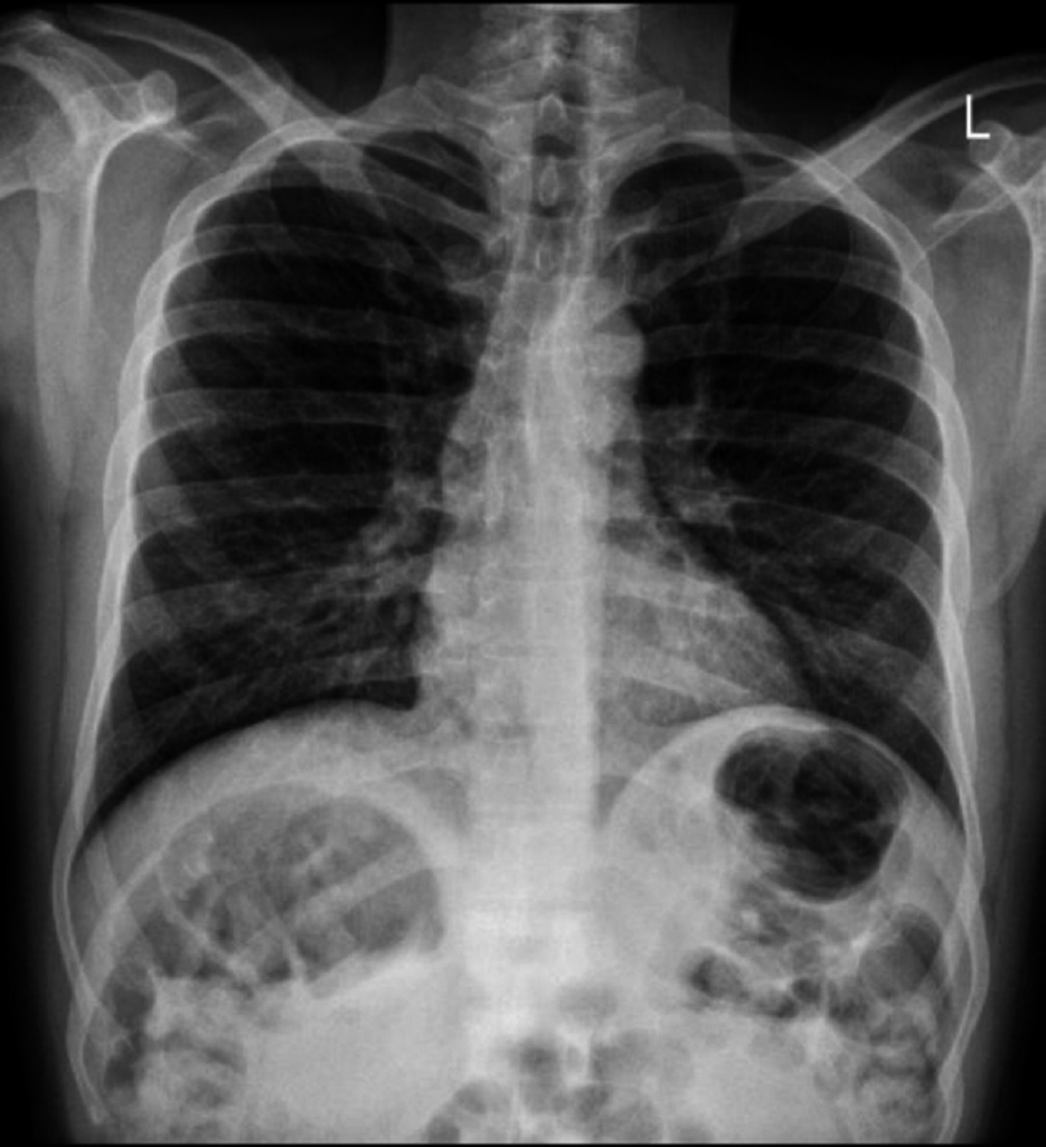
Upright Chest X‐ray

**FIGURE 2:**
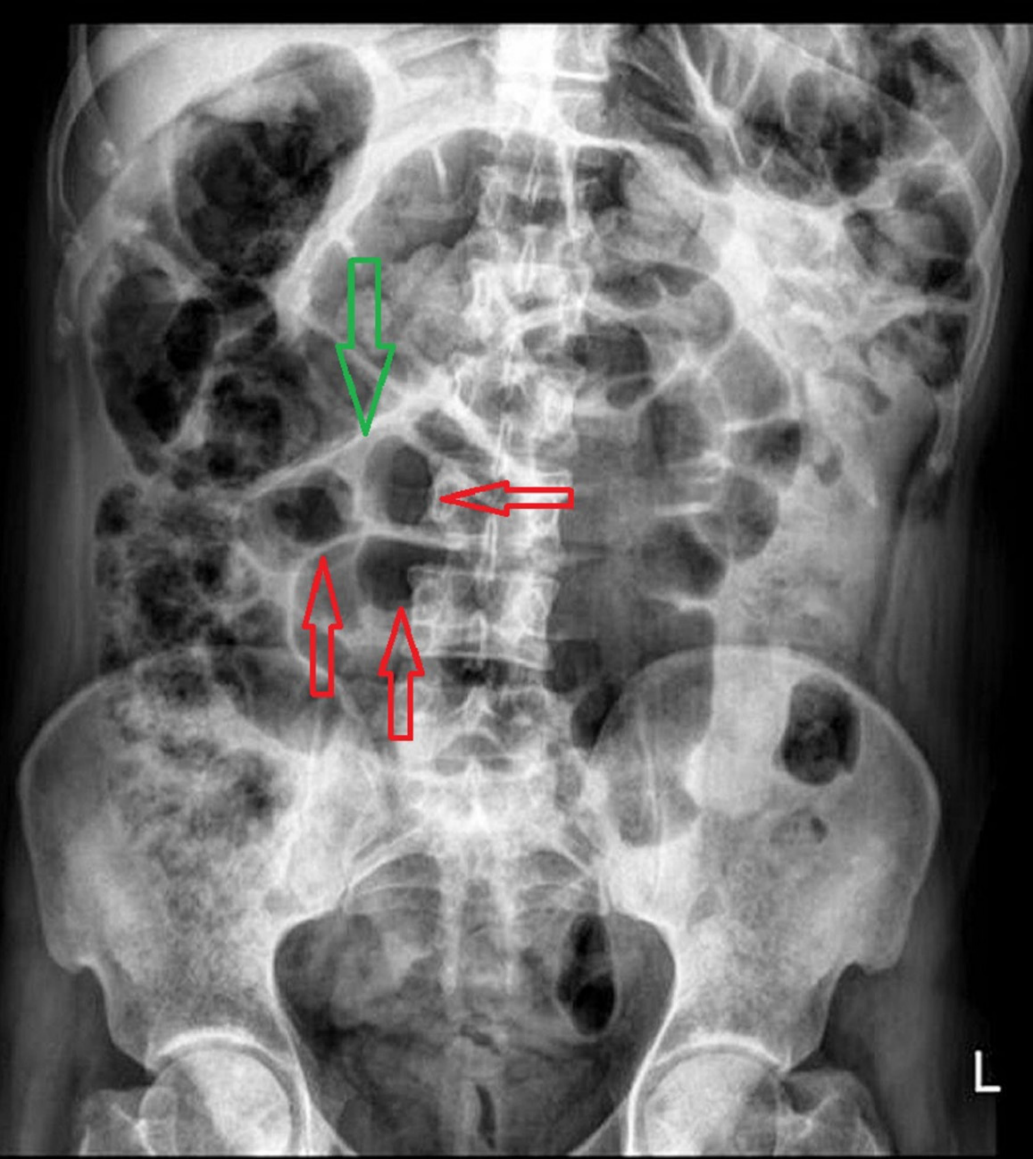
Supine abdominal X‐ray, red arrows show gas‐filled bowel loops, the green arrow points to Rigler sign

**FIGURE 3:**
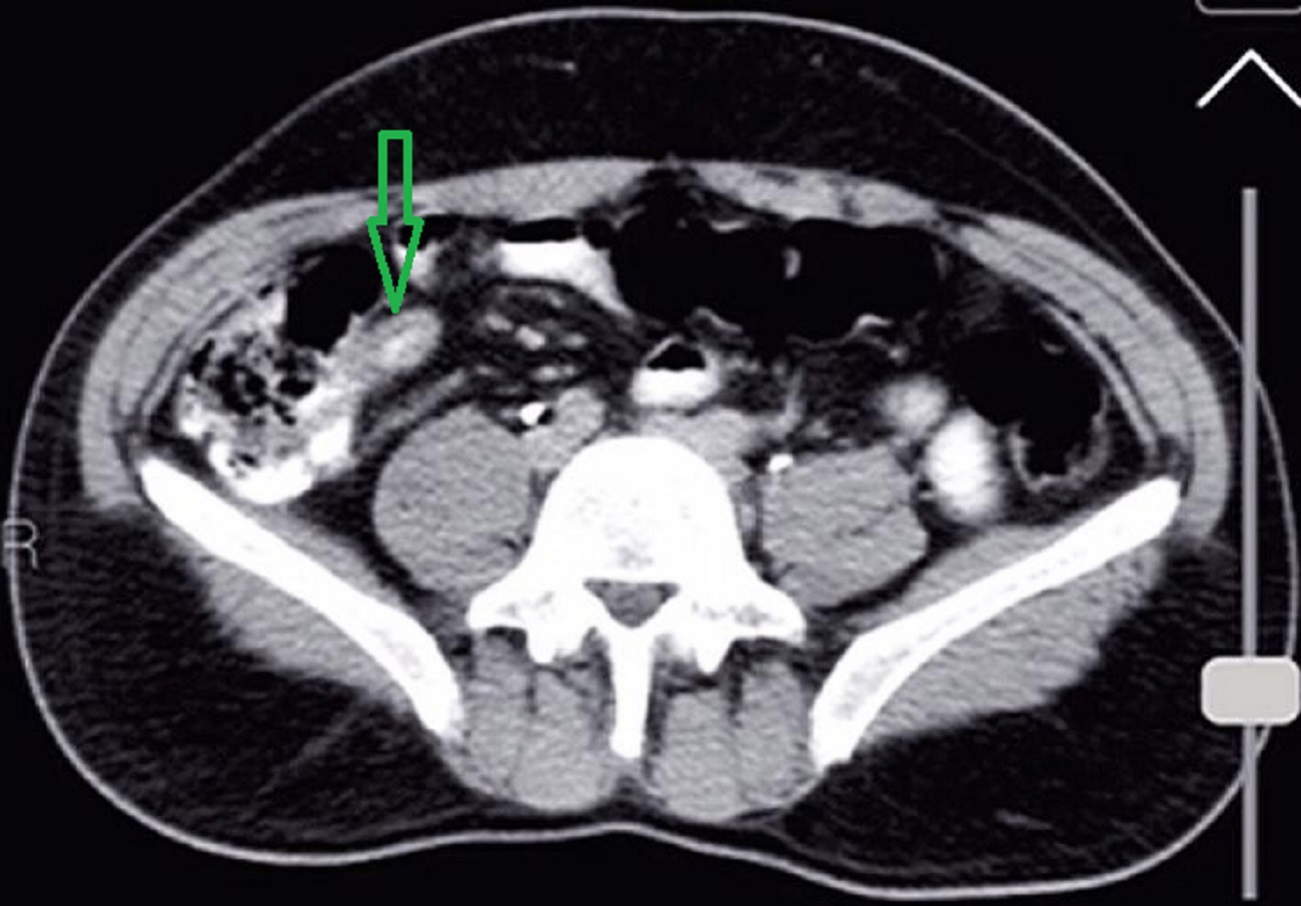
Abdominal CT‐scan, the green arrow shows appendix filled by IV contrast fluid

A 35‐year‐old male patient presented to the surgical department of Poursina Hospital Medical Center, Rasht, Iran, in October 2021, with no history of underlying diseases and with complaint of abdominal pain started 5 days ago with a predominance of the left lower quadrant of the abdomen and hypogastric area and two times vomiting, a day before hospitalization. The patient claimed that the abdominal pain had been vague and generalized and started 5 months ago for the first time. It was persistent but improved with the use of painkillers. The pain was worsened with feeding and relatively relieved by lying supine, and it had nothing to do with defecation and gas passing. The patient was a drug abuser. He also mentioned occasional constipation from the beginning of the pain in the past 5 years ago. Moderate but progressive generalized tenderness was detected during physical examination but guarding, and rebound tenderness were not detected. He had a low‐grade fever, estimated at 38.1 Celsius, but other vital signs were normal. Therefore, due to the patient's symptoms, he was admitted to the surgical ward with suspicion of general peritonitis due to the perforated peptic ulcer. He was asked to do an upright chest X‐ray and supine abdominal X‐ray. The upright chest X‐ray pictures were unrevealing, as shown in Figure 1. As shown in Figure 2, dilated, gas‐filled bowel loops in the supine abdominal X‐ray could signify mechanical or ileus obstruction. Furthermore, the Rigler sign can be seen in the abdominal X‐ray that was a sign of pneumoperitoneum, leading the surgical team to reinforce the suspicion of perforation. He was also asked to do an endoscopic procedure, and the results showed erosive gastropathy and duodenal ulcer, and the patient was prescribed high‐dose proton pump inhibitors (PPI). After that, he was asked to do abdominal sonography. The result revealed a blind loop in the right lower quadrant (RLQ) of the abdominal cavity with standard size in the base and proximal parts. Still, it increased the diameter in the Tip that it was approximately 7 mm. It was non‐compressive and with slight fat haziness around it. These findings led to suspicion of appendicitis. Due to the inconsistency of the clinical findings during the physical examination with the ultrasonography findings and due to the suspicion of perforated or complicated appendicitis, he was advised to do abdominal computed tomography (CT) scan with intravenous and oral contrast for more investigations. The CT‐scan imaging confirmed a mass‐like lesion in the RLQ of the abdominal cavity that could represent appendicitis and tissue wall, thickening in the AP region. Still, unlike usual appendicitis, the IV contrast fluid filled the appendix, as shown in Figure 3. The blood test analyzed presented a standard range of lactate dehydrogenase (LDH) =309 (usually should be under 460 in adults), leukocytosis (white blood cells [WBC] =11200 g/dl with a neutrophilia ratio of 73%), and Amylase =54 U/Lit (normally should be under 95).

**FIGURE 4:**
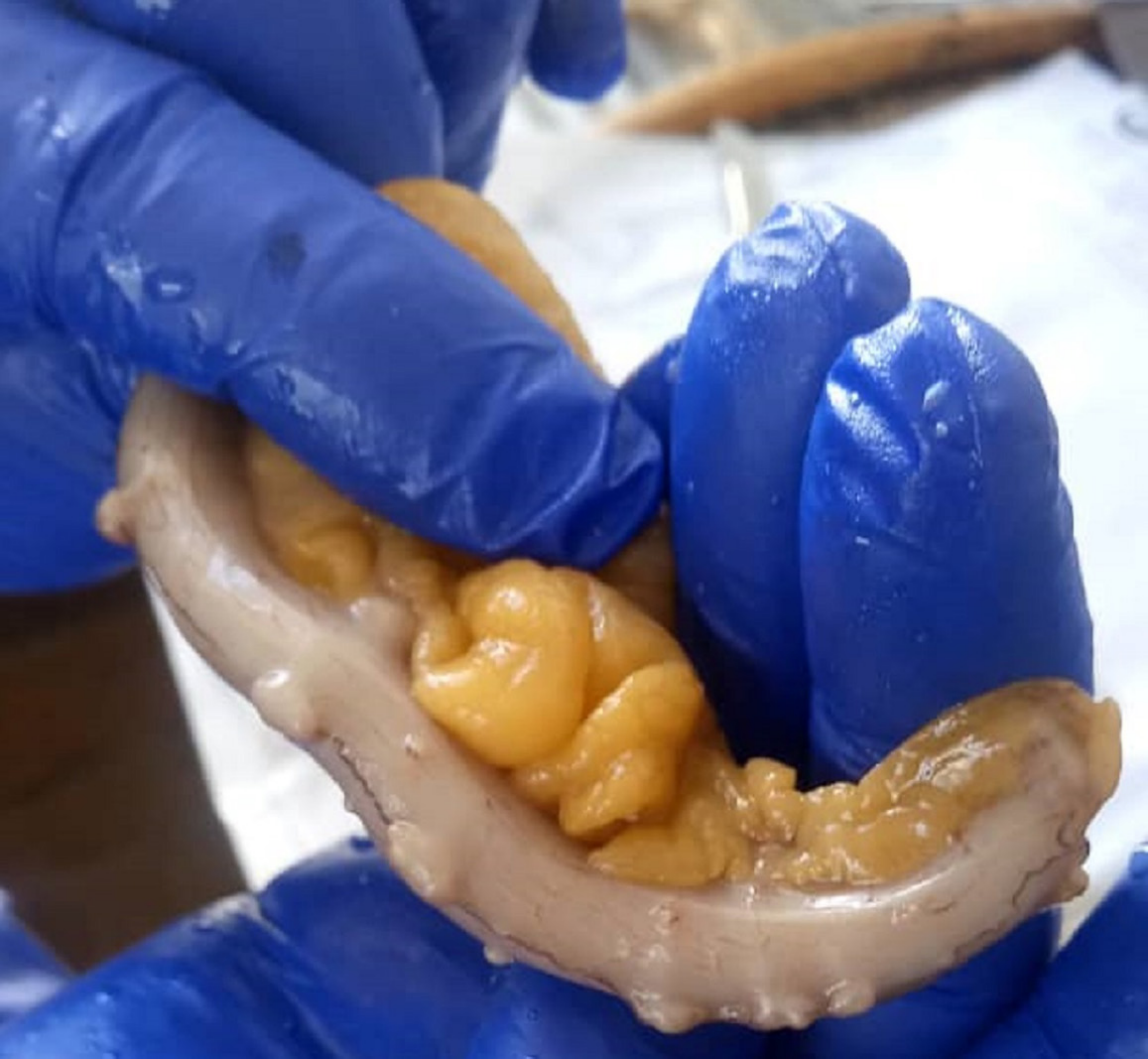
Appendix after appendectomy

**FIGURE 5:**
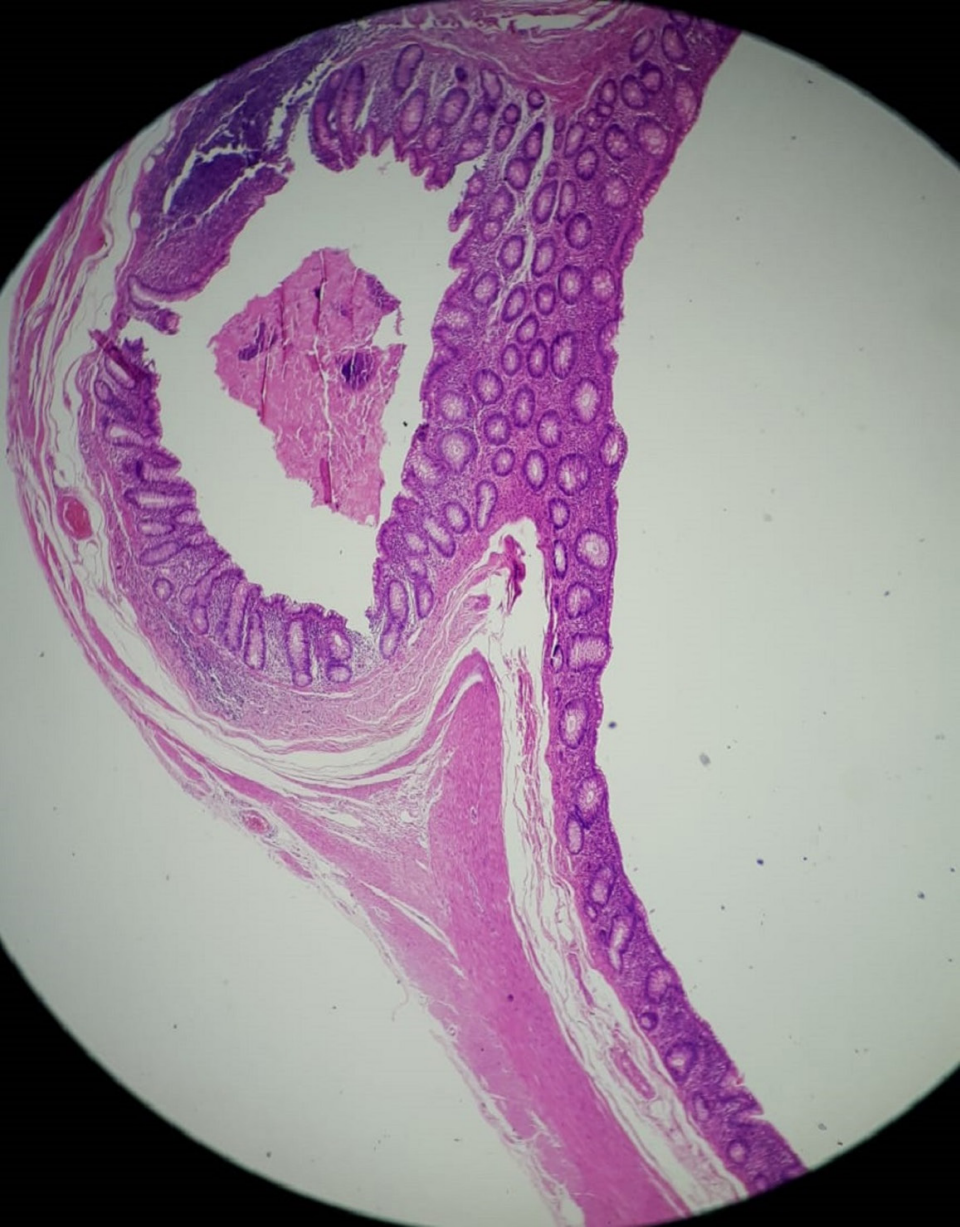
Microscopic view of diverticular appendix tissue (True lumen)

**FIGURE 6:**
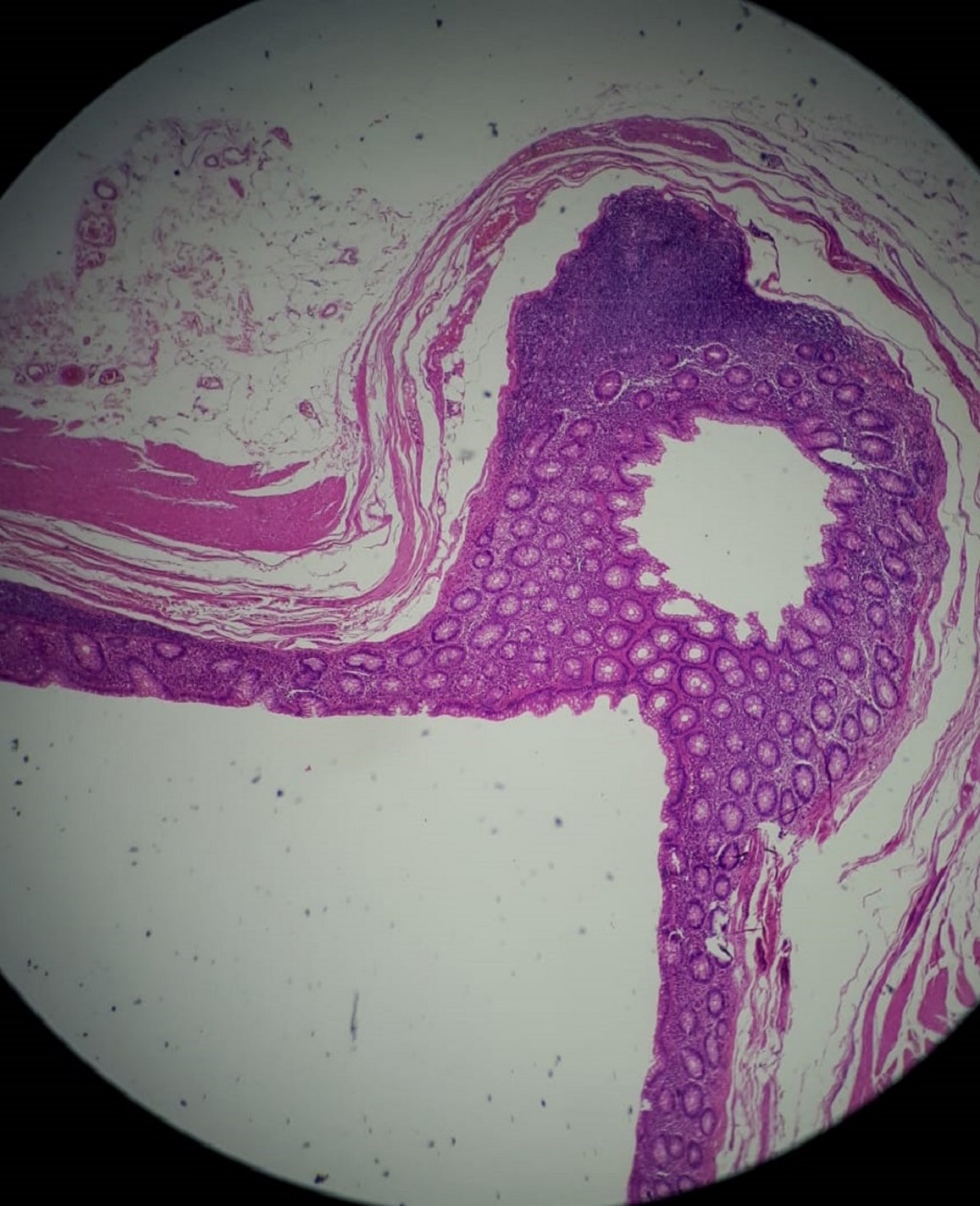
Microscopic view of diverticular appendix tissue (True lumen)

Unfortunately, generalized abdominal tenderness intensified during hospitalization, and hypogastric rebound tenderness was found as a new sign. Therefore, in the second day of hospitalization, the patient underwent laparotomy with suspicion of general peritonitis due to the perforated peptic ulcer or perforated appendicitis. A midline incision was performed. On the external side of the duodenum wall, the stiff tissue was touched at the same site as an ulcer reported by endoscopy but no perforation was detected. Also, a mass‐like lesion was seen in the right lower quadrant area of the abdomen cavity with lots of adhesions to its around tissues. It was discreetly removed from the surrounding tissues, and a diverticular appendix appeared, as can be seen in Figure 4. The tissue was sent for more pathological investigations. After providing the necessary hemostasis, the abdominal cavity was closed. He had a good recovery, and the vital signs were stable after surgery. The patient was transferred to the ICU ward and transferred to the surgical ward after 1 day and discharged after 4 days with good general condition and stable vital signs. Ciprofloxacin and Metronidazole treatment was started for him and continued for 7 days. There was no complication in the three‐month follow‐up. The pathological reporting showed herniation of mucosa and submucosa and muscular layer through the wall of appendix and confirmed the diagnosis of the appendix with multiple diverticulosis and appendiceal diverticulitis, as can be seen in Figures 5 and 6.

## 3 DISCUSSION

Different cases of appendiceal diverticula with different symptoms and complications have been reported, including patients of various ages and with different health conditions.^2^, ^6^, ^7^ This disease is classified into two groups based on the number of layers herniating through the appendix wall. Acquired or pseudodiverticula form and congenital or true form, which means all three appendiceal layers herniate through a normal wall histologically.^2^, ^4^, ^8^ The congenital type is rare and accounts for 3% of all diagnosed appendix diverticulosis cases.^9^, ^10^, ^11^ Appendiceal diverticulitis risk factors are male gender, age over 30, cystic fibrosis, and Hirschsprung disease.^12^ Appendiceal diverticulitis is not only a rare condition that can mimic other diseases’ symptoms, and most of all, it is confused with acute appendicitis, but also it can be synchronous with other serious diseases such as carcinoid tumors.^2^, ^5^ One of the most complications of appendiceal diverticulitis is perforation, with an incidence prevalence of 66%.^13^ Other complications could be chronic pain and acute inflammation.^14^ Due to these reasons, it is crucial to diagnose this problem preoperatively. Using imaging methods such as ultrasonography and CT‐scan can be very useful to diagnose appendiceal diverticulitis, while CT‐scan is better and has 80% sensitivity and 100% specificity. Still, both methods are highly dependent on radiologists’ experiences.^10^, ^13^ On the contrary, the diagnosis of appendiceal diverticulitis may not be possible due to the small size or involvement of inflammatory mass.^12^ So, the definitive diagnosis way is a postoperative pathology report.^15^

The definitive treatment way to eradicate symptomatic appendiceal diverticulitis is an appendectomy, and choosing the appropriate surgical method between laparotomy and laparoscopy depends on the patient's condition and surgical team decision.^15^ Choosing the appropriate surgical method is crucial to perform a safe way to avoid rupture that it can lead to peritoneal seeding and peritonitis consequently.^5^

Valentino's syndrome should be considered as an important differential diagnosis in this situation. It is a life‐threatening condition that occurs by gastric or duodenal fluid collection in the right paracolic gutter and leads to focal peritonitis and right lower quadrant pain.^16^

In this article, the male patient presented with abdominal pain and generalized abdominal tenderness with suspicion of perforated peptic ulcer. After medical investigation, such as physical examination, blood test analysis, ultrasonography, and endoscopy procedure, the patient was diagnosed with a non‐perforated duodenum ulcer and appendicitis. These diagnoses justified the patient's symptoms as the simultaneous occurrence of peptic ulcer and appendicitis can cause generalized abdominal pain and generalized abdominal tenderness. However, as a rebound tenderness suddenly appeared in the physical examination and the patent's abdominal tenderness progressed from moderate to severe, the surgical team decided to choose the laparotomy method instead of the laparoscopy one due to suspicion of general peritonitis and the patient's condition.

## 4 CONCLUSION

This article is about a 35‐year‐old male patient presenting with generalized abdominal pain with a predominance of the left lower quadrant of the abdomen and hypogastric area mimicking perforated peptic ulcer but found to have abdominal peritonitis due to appendiceal diverticulitis. It is crucial to diagnose and treat this disease preoperatively as some studies showed that appendiceal diverticulitis could be asymptomatic until getting infected or accidentally during a medical investigation or can mimic other diseases’ symptoms or occur simultaneously with other serious diseases. So, using different imaging methods such as ultrasonography and CT‐scan could be beneficial, but physical examination findings should be considered too. However, the definitive way to diagnose the disease is postoperative pathological investigation.

Therefore, appendiceal diverticulitis should be regarded as an important differential diagnosis in patients with abdominal pain.

## CONFLICT OF INTEREST

The authors certify that there is no conflict of interest with any financial organization regarding the material discussed in the manuscript. The patient has consented to the submission of the case report for submission to the journal.

## AUTHOR CONTRIBUTIONS

All authors contributed equally to the manuscript and read and approved the final version of the manuscript.

## CONSENT

Written informed consent was obtained from the patient to publish this report in accordance with the journal's patient consent policy.

## Data Availability

The data that support the findings of this study are available on request from the corresponding author. The data are not publicly available due to privacy or ethical restrictions.
